# Severity of Chest Pain among Acute Myocardial Infarction Patients with Diagonal Branch Vessel Disease: A Pilot Study

**DOI:** 10.7759/cureus.5519

**Published:** 2019-08-29

**Authors:** Muhammad Nasir Rahman, Azmina Artani, Farhala Baloch, Bilal Hussain

**Affiliations:** 1 Cardiology, The Aga Khan University, Karachi, PAK; 2 Epidemiology, The Aga Khan University, Karachi, PAK

**Keywords:** chest pain, diagonal branch vessel disease, acute myocardial infarction

## Abstract

Introduction: Acute myocardial infarction (AMI) patients present with variable clinical manifestations such as shortness of breath, nausea, etc. among which chest pain is the most common. Previous studies have reported that the clinical presentation of AMI patients with branch vessel disease is indistinguishable from epicardial coronary vessel disease. However, our experience suggests patients with branch vessel disease experience severe chest pain, especially those with a diagonal branch. Therefore, we aim to study the association of chest pain severity with isolated diagonal branch vessel disease as a culprit vessel in AMI patients.

Methods: It is a retrospective case-control design, where 10 cases and 40 historic controls were recruited in the study. Cases were patients with isolated diagonal branch disease, whereas controls were patients with epicardial vessel disease in AMI. We reviewed Coronary Angiograms of adult patients who presented with acute myocardial infarction and had undergone coronary angiography at Aga Khan University Hospital, Karachi (AKUH). Information on pain scores was measured using the Numeric Pain Rating Scale (NRS) before administration of analgesics. Other relevant variables were also recorded on a pre-structured questionnaire.

Results: The mean age of all the participants in the study was 60 ± 11.0 years, with 16% of the patients being women. Among all AMI patients, the intensity of chest pain in patients with isolated diagonal branch vessel disease was 2.6 units higher as compared to those with other epicardial coronary vessel diseases (p-value: <0.001; 95% CI: 1.67 - 3.46).

Conclusion: This preliminary study indicates severe chest pain can be a differentiating symptom in AMI patients with diagonal branch disease. It emphasizes clinicians to look for a possibility of a diagonal branch as a culprit vessel in AMI for better judgment, as it is often overlooked. future studies may be conducted at multiple centers for larger sample size and better generalizability.

## Introduction

Acute myocardial infarction (AMI) is one of the leading causes of death worldwide [1]. It results from plaque rupture in coronary arteries. Patients with acute myocardial infarction may experience a range of symptoms such as shortness of breath, sweating, and nausea. Chest pain is considered as one of the classical signs of AMI [2].

Chest pain characteristics such as intensity, onset, relieving and aggravating factors may differ from patient to patient [3]. Many researchers across the globe have contributed their knowledge to this phenomenon and have tried to explain the mechanism behind cardiac pain perception [4]. Despite available literature, there is a limited understanding of exact pain pathways and precipitating factors to pain, such as the intensity of pain with respect to the region of myocardium being affected. Pain intensity may also differ across different regions of the heart because of the variable amount of blood supply [5-6].

In our practice, we often noticed patients with occlusion of a diagonal branch [branch arising from the left anterior descending artery (LAD)] to present with more severe chest pain as compared to patients with occlusion of other major epicardial vessels [left circumflex (LCX) and right coronary artery (RCA)] as infarct-related artery. In these patients, without suggestive findings of occlusion in epicardial vessels, there is a high likelihood of missing the involvement of a diagonal branch as a culprit vessel. In such circumstances, it may lead to a diagnostic dilemma if significant attention is not given while interpreting coronary angiograms. Therefore, in this pilot study, we aimed to study the association of intensity of pain with isolated diagonal branch vessel disease as compared to epicardial vessel disease in AMI patients.

## Materials and methods

Study design and population

The study incorporated a case-control design and was conducted at the Aga Khan University Hospital, Karachi (AKUH). Data was extracted for patients who presented with AMI to the emergency department at AKUH from the period of June 2014 to June 2016. Participants who were ≥ 18 years and had complete medical records were included in the study. We excluded patients who were on mechanical ventilation, had received analgesics prior coming to the emergency department from health care facilities outside AKUH, had collateral circulation to the obstructed diagonal branch or epicardial vessel, and those with known symptomatic neuropathy. Patients who were known opioid addicts were also excluded as they may have altered pain thresholds because of opioid use.

We defined cases as patients who had isolated diagonal branch, without the involvement of LAD or any other major vessels (such as LCX and RCA). Controls were defined as patients whose coronary angiogram revealed other epicardial coronary vessels (LAD (without the involvement of the diagonal branch), LCX, and RCA).

Data collection

We began with the identification of cases during the period of June 2015 to June 2016 by reviewing coronary angiograms of AMI patients brought to the cardiac catheterization lab. Upon ascertainment of eligibility criteria, cases and controls were enrolled in the study. As we identified each case, we randomly selected four historic controls upon reviewing the coronary angiograms of previous AMI patients during the study period.

Ascertaining outcome: pain assessment

Our hospital utilizes the Numeric Pain Rating Scale (NRS) as the most common tool for quantifying pain perception among adults with no cognitive or communication difficulties [7]. The NRS pain scale requires patients to assign a numeric value to the pain from a scale of 0 - 10 where 0 represents no pain and 10 represents maximum pain that they must be having [8].

As a standard of care, upon initial assessment of vital signs, a trained doctor or nurse assesses the pain of all AMI patients using the NRS scale. Electrocardiogram (ECG) of these patients is then performed in order to reach an affirmative diagnosis of AMI. Pain medications are administered to the patients before shifting them to the cardiac catheterization lab for the intervention. These initial assessments are documented in the patient’s progress notes, which become part of the hospital record upon the patient’s discharge. For this study, the maximum pain score of each patient before the administration of analgesics prior to the procedure was recorded. Information on the history of co-morbid conditions and interpretation of ECG at the time of arrival was also noted.

Ethical approval was sought from the Ethics Review Committee, AKU (4358-Car-ERC-16) which granted an exemption for ethical approval and permission to review the hospital records for this study.

Sample size and statistical analysis plan

The prevalence of diagonal branch vessel disease as culprit vessel in AMI is very low in the general population, i.e., 0.5%-1% [9-10]. Therefore, a total sample of 50 patients was demised sufficient employing a case-control design (cases and controls to be selected in a ratio of 1:4). We assumed a 35% of severe pain exposure among controls [11-12], to achieve a minimum power of 80% and 95% confidence level. The sample size was calculated using OpenEpi Software.

For continuous variables, we report means and standard deviations for normally distributed variables, whereas, for non-normally distributed variables, we report median and interquartile range. For categorical variables, we determined frequencies and proportions. To examine the difference in baseline characteristics of quantitative variables, t-test for an independent sample was applied. Chi-square or Fisher Exact test were applied for categorical variables, as appropriate. A multiple linear regression was performed to determine the association of pain intensity between cases and controls keeping pain score as an outcome, while controlling for other factors and comorbidity profile of participants. Confounding and interaction were also assessed for biologically plausible variables. Data were analyzed using STATA version 12. A p-value of ≤ 0.05 was deemed significant.

## Results

A total of 50 participants (10 cases and 40 controls) were included in the study upon review of the records. Among controls, 55% of the patients had LAD, 25% had left circumflex and 20% had RCA as culprit vessel in AMI. As the continuous variables in the data followed a symmetrical distribution, we report means ± standard deviation here. Cases and controls had a similar distribution of characteristics with respect to age, gender, co-morbid conditions (diabetes, dyslipidemia and family history of coronary artery disease (CAD)) and smoking status. However, more of the patients had been hypertensive in the control group (Table 1). The mean pain score among the cases was 9 ± 1.1 as compared to that of 6 ± 1.1 among controls and was statistically significant (t-test p-value <0.001). 

**Table 1:** Baseline Characteristics of Cases and Controls among Acute Myocardial Infarction Patients SD = Standard Deviation

Variables	Cases (n=10)	Controls (n=40)	P-value for Difference
Age in years (Mean, ± SD)	60.1 (10.7)	59.8 (11.7)	0.92
Female gender (n, %)	3 (30)	5 (12.5)	0.33
Hypertension (n, %)	4 (40)	35 (87.5)	0.004
Diabetes (n, %)	5 (50)	24 (60)	0.56
Dyslipidemia (n, %)	2 (20)	19 (47.5)	0.16
Current Smokers (n, %)	5 (50)	13 (32.5)	0.30
Family history of coronary artery disease (n, %)	2 (20)	2 (5)	0.17

For linear regression, scale examinations of the continuous variables (age and numeric pain scale) were done which revealed no difference in the value of R^2^ at the univariate level. Hence, they were kept as a continuous variable for the multivariable linear regression model. A stepwise method was opted for multivariable modeling to build a parsimonious model with maximum R^2^. The intensity of pain was 2.6 units higher among patients with diagonal branch vessel disease (cases) as compared to those with epicardial vessel disease (controls) [95% CI: 1.67 - 3.46], adjusted for all other covariates in the model (Table 2). Confounding and interaction were checked for diabetes, hypertension, gender, and age but was not found significant. 

**Table 2:** Association between Intensity of Pain and Diagonal Branch Vessel Disease among Acute Myocardial Infarction Patients CAD = Coronary Artery Disease

Variables	Adjusted β-coefficient	95% Confidence Interval	p-value
Diagonal branch vessel (cases)	2.6	1.67 – 3.46	<0.001
Family history of CAD	1.2	-0.06 – 2.4	0.06
Age (years)	- 0.01	-0.04 – 0.1	0.2
Hypertension	0.46	-0.48 – 1.4	0.33
Type II Diabetes	- 0.2	-0.86 – 0.44	0.52

Pain perception of patients with a family history of coronary artery disease was slightly higher (adjusted β-coefficient: 1.2), however, the difference was marginally statistically insignificant (p-value: 0.06). There was no association of pain intensity with age, hypertension, and diabetes. Future studies may look into a larger sample population from multiple centers to explore the impact of family history and knowledge about the symptoms of AMI among these patients.

## Discussion

This preliminary study highlights a distinct clinical feature of acute myocardial infarction patients with diagonal branch as an infarct-related artery. We observed AMI patients present with severe chest pain without evidence of disease in any major epicardial vessels on the coronary angiogram in our practice. On the contrary, we found disease in one of the branch of major epicardial vessels, which was mostly a diagonal branch causing severe pain. From the review of the existing literature, we know that the severity of chest pain per se does not correlate with the severity of CAD [13]. In addition, patients with isolated branch vessel disease have clinical presentations that are indistinguishable from those of patients with coronary disease involving the major vessels [9,14]. Evidence regarding the association of pain severity and diagonal branch vessel is very limited and has not been studied clinically. Hence, we decided to test this hypothesis in this pilot study.

Our preliminary data confirms our observation that patients with diagonal branch vessel CAD have more severe chest pain in the condition of AMI. One of the possible pathophysiologies can be explained by the diameter of the vessel. The delivery of oxygen-rich blood to the myocardium is influenced by coronary artery diameter, the presence of collaterals and perfusion pressure determined by the pressure gradient from the aorta to the coronary arteries [15]. Due to a smaller diameter compared to the epicardial arteries, a branch vessel can have persistent vasomotor tone during coronary ischemia, resulting in severe pain due to lack of vasodilators [16]. This phenomenon may also be true for other branch vessel disease however; it was beyond the scope of this study. In addition, the diameter of the vessel has some relation with coronary auto regulatory mechanism during coronary hypo perfusion. Studies in the past have shown that the magnitude of dilatation during coronary hypo perfusion is inversely related to the initial diameter of the vessel [16]. However, it is yet to be known whether the severity of pain is due to the difference in the distribution of pain receptors in different areas of the heart and will serve basis for basic sciences studies in the future [17].

The strength of our study is that it highlights the evidence on the association of pain and diagonal branch vessel disease emphasizing on possible mechanisms between pain pathways and ischemic territories being sensitive to particular branches or vessels. However, the study has certain limitations inherent to the design of retrospective studies and cannot determine causality. As the condition under study is less common (AMI patients with a diagonal branch as culprit vessel), the single-center study contributed to a limited number of cases, while a random selection of controls permitted us to limit selection bias. Future studies involving multiple centers may make this primitive population more accessible for exploration and may be proposed to observe a difference in pain perception between the two groups incorporating knowledge of AMI symptoms and total ischemic time.

## Conclusions

To conclude, our research suggests that if patients present with severe chest pain without significant disease in the major epicardial vessels, one should always look for disease in the diagonal branch vessel, especially in the absence of significant ECG changes. A more aggressive strategy towards the management of chest pain in patients with diagonal branch vessel disease may be required and may lead to higher patient satisfaction and comfort.
